# C-Terminal Regions of Insecticidal Cry Toxins Form Amyloid Fibrils Affecting Metabolism of Insect Cells

**DOI:** 10.3390/ijms27198654

**Published:** 2026-09-28

**Authors:** Haidar Fayoud, Anna V. Kondrateva, Mikhail V. Belousov, Anton E. Shikov, Alexander G. Bobylev, Kirill V. Smirnov, Anton A. Nizhnikov, Kirill S. Antonets

**Affiliations:** 1 All-Russia Research Institute for Agricultural Microbiology, 196608 St. Petersburg, Russia; 2 Faculty of Biology, St. Petersburg State University, 199034 St. Petersburg, Russia; 3 Institute of Theoretical and Experimental Biophysics, Russian Academy of Sciences, 142290 Pushchino, Russia

**Keywords:** amyloid, fibril, aggregate, crystal toxin, Cry, bacteria, *Bacillus thuringiensis*, *Bt*

## Abstract

Crystal (Cry) toxins produced by *Bacillus thuringiensis* are highly specific insecticidal proteins that are widely used as biological insecticides and function through proteolytic activation in insect midguts, which generates a three-domain toxic core responsible for membrane disruption as well as cleaved N- and C-terminal domains. While the structure and function of this core have been extensively characterized, the fate of the cleaved C-terminal region remains poorly understood. Amyloids are protein fibrils with a characteristic cross-β structure that participate in a wide range of functions in bacteria. Here, using bioinformatic approaches, we demonstrated that the C-terminal domains of Cry are rich in potentially amyloidogenic regions. We investigated the aggregation behavior of C-terminal domains from Cry1Ea11 and Cry1Ab12 proteins using a combination of biochemical and structural approaches. We found that the C-terminal domains of Cry1Ea11 and Cry1Ab12 undergo self-assembly in vitro, forming aggregates with fibrillar morphology. These assemblies bind amyloid-associated dyes, exhibit partial resistance to ionic detergents and proteolytic digestion, and display X-ray diffraction patterns confirming their amyloid properties. Functional assessment in insect cells showed that although these amyloids do not induce detectable cytotoxicity, amyloids of the C-terminal domains of Cry1Ea11 alter the readouts of the MTT assay in a manner consistent with previously described amyloid-associated effects on cellular formazan handling. Together, our findings demonstrate that the C-terminal domains of Cry toxins, which are released in the insect midgut as a result of proteolytic cleavage, are able to form bona fide amyloids that affect the metabolism of insect cells.

## 1. Introduction

*Bacillus thuringiensis* (*Bt*) is a Gram-positive, spore-forming bacterium characterized by its ability to produce proteinaceous crystalline inclusions during its sporulation phase [1]. Owing to their high specificity for target insect pests, *Bt* toxins have been extensively utilized in commercial biopesticides [2,3]. Furthermore, genes encoding *Bt* toxins have been widely introduced into genetically engineered plants, known as *Bt* crops, conferring enhanced resistance to insect herbivory [4,5,6].

While *Bt* produces up to six distinct groups of crystal-forming toxins [7], the crystal (Cry) toxins remain the most extensively characterized and widely applied [8,9]. Cry toxins share a conserved three-dimensional architecture comprising three functional domains [10]. Each domain plays a specific role in the infection process: domain I is primarily involved in membrane pore formation and subsequent cell lysis, whereas domains II and III mediate receptor recognition and binding to the insect midgut epithelium [11]. Following oral ingestion, Cry protoxins dissolve in the alkaline gut lumen and undergo activation via proteolytic cleavage mediated by insect digestive enzymes [12]. The activated toxins then bind to epithelial receptors, inducing toxin oligomerization and the formation of transmembrane pores [13]. These pores disrupt cellular osmotic balance, leading to cell lysis and permitting *Bt* to invade the nutrient-rich hemocoel. The resulting septicemia ultimately causes insect death [14]. However, proteolytic activation not only generates the active toxic core but also releases terminal fragments whose structural and functional fate remains poorly understood. Previous studies have shown that the N-terminal region of the toxin contributes to specificity and activation efficiency [15,16,17]. In contrast, the fate and potential functional relevance of the C-terminal region following proteolytic cleavage remain largely unexplored. Structural studies indicate that the C-terminal domains can form intra- and intermolecular bonds that contribute to the formation and stabilization of the crystal [18]. Notably, structural analyses have indicated that the C-terminal region in certain Cry toxins is enriched in β-sheet structures [19]. Upon proteolytic release, such β-rich structural elements may possess an intrinsic propensity for self-association and ordered aggregation, raising the intriguing hypothesis that they could assemble into amyloid structures.

Amyloids represent a distinct class of protein fibrillar aggregates defined by a characteristic cross-β architecture conferring high structural stability [20,21,22,23,24,25]. Hallmark features of amyloids include the characteristic cross-β pattern observed via X-ray diffraction [26], apple-green birefringence under polarized light upon Congo Red (CR) staining [27], and increased fluorescence of bound Thioflavin T (ThT) [28]. While historically associated with neurodegenerative pathologies, amyloid structures are now recognized as widespread and functional across all three domains of life [22,29,30]. In bacteria, functional amyloids perform a wide spectrum of functions, including some aspects of ensuring interaction between bacteria and a multicellular host. In particular, a set of proteins from different bacterial species form amyloids that play key roles in biofilm formation and maintenance, metabolism of bacterial toxins, and modulation of surface properties [31,32,33]. The most studied and first discovered example of functional bacterial amyloids is curli [34], predominantly formed by CsgA in *Escherichia coli* and some other enterobacterial species. Curli amyloid fibrils are a major component of uropathogenic *E. coli* biofilm extracellular matrix comprising about 85% of its weight [35]. Thus, functional bacterial amyloids are important contributors to pathogen–host interactions.

Despite the increasing recognition of functional bacterial amyloids, it remains unknown whether C-terminal regions of Cry toxins possess intrinsic amyloidogenic potential after protoxin processing. This question is conceptually distinct from the study of full-length bacterial amyloid proteins because Cry C-terminal regions correspond to fragments generated during proteolytic activation, a central step in the mode of action of Cry protoxins. Here, we combine bioinformatic predictions with biochemical and structural analyses to investigate the amyloidogenic properties of C-terminal domains from Cry1Ea11 and Cry1Ab12. We show that these domains can form ordered amyloid assemblies in vitro. By addressing the structural fate of C-terminal regions released during Cry toxin activation, this study expands the current understanding of Cry toxin structural plasticity and highlights the potential biological relevance of proteolytically generated toxin fragments.

## 2. Results

### 2.1. In Silico Analysis of the Available Cry Toxins Reveals High Amyloidogenic Potential of C-Terminal Domains

To carry out computational predictions of amyloid properties in Cry toxins, we first retrieved all available reference sequences from the BPPRC (Bacterial Pesticidal Protein Resource Center) database [36] and homologous proteins from the GenBank resource [37] and predicted potentially amyloidogenic regions using the Waltz [38,39] and SARP [40] algorithms. From a total of 1264 toxins, 1061 representatives (84%) contained at least one amyloidogenic region (Figure 1A). On the whole, proteins contained from one to ten zones with an average of two sites (Figure 1A). The distribution of total protein-wise lengths of predicted amyloidogenic regions exhibited a high dispersion, ranging from 785 to 11 amino acid residues; however, the former seems to be an overestimation, given that the median reached 23 residues (Figure 1A). Individual sites belonging to the C-terminal domain were slightly yet significantly (*p* < 2 × 10^−16^) longer than those associated with other domains (14.4 vs. 13.4, respectively) (Figure 1A).

Of all the identified individual sites, 604 out of 2179 (28%) were localized within the C-terminal domains and were distributed across all structural domains of the toxins (Figure 1B). Notably, the overall coordinate-wise distribution along the sequences was generally uniform. Nevertheless, we observed an intriguing pattern with two relatively small (circa 11 aa residues) signals at the beginning of the first domain and in the middle of the C-terminal region (Figure 1B). Since the latter is placed outside the dense three-domain functional complex, one might expect this region to play a role in amyloid formation. Notably, we revealed that this region is present in Cry toxins deposited in our collection, namely, Cry1Ab12 and Cry1Ea11 (Figure 1C).

Therefore, bioinformatic analysis of the Cry toxins diversity points to the ability to form aggregates, which is supported by the abundance of amyloidogenic motifs distributed across several regions, including the C-terminal domains. However, bioinformatic analysis per se does not prove the biochemical properties. Thus, we proceeded to experimental verification to disentangle whether the C-terminal domains of Cry toxins could organize into amyloid structures.

### 2.2. C-Terminal Domains of Cry Toxins Form Fibrillar Aggregates In Vitro

To evaluate the fibril-forming ability of the analyzed proteins in vitro, recombinant C-terminal domains of Cry1Ea11 (Cry1Ea11C) and Cry1Ab12 (Cry1Ab12C) were produced in *E. coli* and purified. Following prolonged incubation under fibrillogenesis conditions (see Section 4), samples were analyzed using transmission electron microscopy (TEM).

TEM analysis revealed that both Cry1Ab12C (Figure 2A) and Cry1Ea11C (Figure 2B) form fibrillar assemblies distributed across the grid surface. Cry1Ea11C (Figure 2B) exhibited more structured aggregates with more pronounced fibrillar morphology. Fibrils formed by both proteins tend to form clusters. The fibrillar structures of the Cry1Ab12 protein had a width of 10.48 ± 2.32 nm, while for the Cry1Ea11 protein it was 16.14 ± 2.8247 nm. Overall, both proteins formed aggregates with fibrillar morphology; however, Cry1Ea11C fibrils were more structured.

### 2.3. In Vitro-Formed Aggregates of the Cry C-Terminal Domains Bind Amyloid-Associated Dyes

Next, aggregates formed in vitro by the C-terminal domains of Cry1Ab12 and Cry1Ea11 were analyzed using Congo Red (CR), Thioflavin T (ThT), and Amytracker staining. The amyloid-forming Sup35NM and the soluble Sup35M proteins were used as positive and negative controls, respectively [41].

The analysis performed demonstrated that both Cry1Ab12C and Cry1Ea11C aggregates exhibited red staining in transmitted light following CR treatment (Figure 3A). Under polarized light, green birefringence consistent with cross-β architecture was observed in both samples (Figure 3B).

In addition, fluorescence analysis using ThT (Figure 3C) and Amytracker (Figure 3D) dyes demonstrated a strong signal enhancement in both samples. Such spectral changes are commonly associated with binding to β-rich aggregated structures, including amyloid assemblies [22,42,43]. Notably, the fluorescence of Amytracker was more pronounced in the case of Cry1Ea11C aggregates, agreeing with their more structured morphology in comparison with Cry1Ab12C. Collectively, these results indicate that the aggregates formed by both C-terminal domains exhibit features typical for amyloid fibrils.

### 2.4. X-Ray Diffraction Reveals Cross-β Features of the Fibrils Formed by the Cry C-Terminal Domains

X-ray diffraction analysis was performed to characterize the structural features of the Cry1Ab12 and Cry1Ea11 C-terminal domain aggregates (Figure 4). The diffraction patterns of both Cry1Ab12C and Cry1Ea11C exhibit distinct reflections in the 4.7 Å range, corresponding to inter-strand spacing within β-sheets, and in the ~10 Å range corresponding to inter-sheet spacing along the fibril axis [26]. These features are characteristic of cross-β structure, which is a hallmark of amyloid fibrils [26], and support the formation of amyloid fibrils by the Cry1Ab12 and Cry1Ea11 C-terminal domains.

Overall, these data indicate that the aggregates formed by C-terminal domains of Cry toxins exhibit a cross-β structure typical for bona fide amyloids.

### 2.5. Aggregates of the Cry C-Terminal Domains Obtained In Vitro Exhibit Detergent and Protease Resistance

To assess aggregate stability, samples were analyzed by SDS-PAGE followed by Western blotting (Figure 5A). At the initial point of incubation, both proteins appeared predominantly in soluble form. Upon incubation, both Cry1Ea11C and Cry1Ab12C exhibited progressive aggregation, reflected by the appearance of high-molecular-weight polymeric species resistant to room temperature (Cry1Ea11C and Cry1Ab12C) and hot (Cry1Ea11C) sodium dodecyl sulfate (SDS) treatment, accompanied by heterogeneous banding patterns. In particular, boiling before electrophoresis reduced high-molecular-weight smearing and increased the intensity of lower-molecular-weight bands, indicating partial disassembly of aggregates (Figure 5A).

Protease digestion with trypsin and proteinase K resulted in the persistence of a protease-resistant species, which shifted upon boiling to lower molecular weight forms (Figure 5A). This behavior is consistent with the presence of compact, protease-resistant cores, a feature commonly associated with the amyloid aggregate formation [22]. Overall, the observed detergent and protease resistance additionally support the formation of bona fide amyloid state by both C-terminal domains.

### 2.6. Cry1Ea11C Amyloids Alter MTT Assay Readouts in Insect Cells

The effects of Cry1Ea11C and Cry1Ab12C amyloid aggregates on insect cell viability were assessed using the MTT assay. Initial assessment of this assay revealed unexpected results. First, treatment of insect cells with Cry1Ea11C and Cry1Ab12C amyloids did not cause a decrease in cell viability (Figure 5B). Second, after two hours of incubation, wells treated with Cry1Ea11C fibrils developed a pronounced blue coloration prior to solubilization, whereas control wells and those treated with non-fibrillar protein remained yellow (Figure 5C). In standard MTT assays, this color change reflects reduction of tetrazolium salt to formazan by metabolically active cells [44,45]. The observed enhancement of signal in treated wells suggested either increased metabolic activity or a potential assay artifact. To determine whether this effect resulted from direct chemical reduction of MTT by the fibrils, cell-free control experiments were performed. No color change was observed under these conditions, indicating that the fibrils do not directly reduce MTT. Therefore, the observed effect depends on the presence of living cells and the fibrillar state of the protein rather than on direct chemical reduction of MTT by the aggregates themselves. To independently validate these findings, a Trypan Blue exclusion assay was performed for control cells and cells treated with Cry1Ea11C samples. No significant differences were observed in live cell counts, dead cell counts, total cell number, or cell viability between fibrillar, non-fibrillar, and control conditions (*p* > 0.05). Cell viability remained within the range of 87–93% across all experimental conditions, indicating that the increased MTT signal observed in Cry1Ea11C-treated cells was not accompanied by detectable changes in cell viability. Taken together, these results indicate that Cry1Ea11C amyloids do not affect insect cell viability but alter MTT assay readouts under the tested conditions.

## 3. Discussion

Cry toxins of *B. thuringiensis* are activated in the insect midgut, generating a functional toxic core [10], while the fate of the released C-terminal region has remained largely unexplored until now. Our findings show for the first time that this domain is not merely an inert fragment but instead possesses an intrinsic propensity to adopt a stable, bona fide amyloid state.

Bioinformatic analysis revealed that amyloidogenic regions are widespread across Cry toxins and are significantly associated with the C-terminal domain, suggesting a conserved aggregation-prone sequence architecture. Importantly, the predicted amyloidogenic motifs (Figure 1B) include a prominent site within the C-terminal domains, supporting a mechanistic link between the in silico predictions and experimentally observed fibrillogenesis. Consistent with this, experimental data demonstrate that isolated C-terminal domains undergo fibrillogenesis in vitro, forming fibrillar aggregates with the cross-β structure that bind amyloid-specific dyes and exhibit resistance to SDS and proteolytic digestion (Figure 2, Figure 3, Figure 4 and Figure 5), indicating that they share key physicochemical features of amyloids.

A particularly striking aspect of this study is the pronounced and enhanced colorimetric signal observed in the MTT assay for cells treated with Cry1Ea11C fibrils (Figure 5B,C). Initially, the results suggested an apparent increase in metabolic activity; however, this interpretation is inconsistent with both the absence of differences in cell viability, live cell counts, dead cell counts, or total cell number confirmed by Trypan Blue exclusion. Instead, this phenomenon is consistent with previously reported amyloid-associated effects on formazan trafficking rather than a true increase in metabolic activity. In particular, it has been established that different mammalian amyloids, including β-amyloid, insulin, and α-synuclein, can interact with the plasma membrane and modulate vesicular trafficking pathways, thereby accelerating the extrusion of intracellularly formed formazan crystals, all of which induce distortions in MTT readouts [46] similar to those reported in our study for amyloids of the Cry1Ea11 C-terminal region. Consistent with this interpretation, the absence of direct MTT reduction in cell-free controls confirms that the observed effect is not due to intrinsic redox activity of the fibrils, as can occur with certain antioxidants or redox-active structures capable of reducing tetrazolium salts [47], but rather arises from their interaction with living cells and the fibrillar state of the protein. Taken together, these results indicate that Cry1Ea11C amyloids do not increase insect cell viability but instead interfere with MTT assay readout, likely through amyloid-associated effects on cellular processes.

Amyloid formation is widely documented in bacteria and studied in more detail in Gram-negative species, where functional amyloids such as curli fibrils not only play central roles in biofilm formation and surface adhesion [34] but contribute to bacterial infection, inflammation, and neurodegeneration in multicellular hosts [48,49,50]. There is multiple evidence of extracellularly located amyloids identified in Gram-negative species [51,52,53] and in Gram-positive species [54,55,56]. All these amyloids are likely to perform mostly structural functions, participating in biofilm formation and thus modulating bacteria-host interactions.

Beyond structural roles, amyloid formation has also emerged as a regulatory mechanism in certain bacterial toxins like listeriolysin LLO of *Listeria monocytogenes*, Microcin E492 of *Klebsiella pneumoniae*, and phenol-soluble modulins of *Staphylococcus aureus*. Moreover, the amyloid state of some toxins can be functionally active, while amyloid formation by other toxins can be used by bacteria to inactivate or store them [57]. For instance, LLO of *L*. *monocytogenes* can undergo conformational transitions under acidic conditions that promote amyloid assembly, modulating cytolytic activity [24,58]. Such environmentally triggered transitions between soluble and aggregated states are a recurring theme among amyloid proteins and may be relevant to the behavior of the Cry toxin C-terminal domains following proteolytic release in the insect midgut. Notably, most described bacterial amyloids involve full-length proteins undergoing regulated assembly. In contrast, examples of physiologically relevant bacterial protein fragments forming amyloidogenic structures remain limited. In this context, the C-terminal domains of Cry toxins represent a distinct case, as they are naturally released through proteolytic activation of the protoxin, raising the possibility that such fragments may possess intrinsic amyloidogenic potential in vivo rather than being simple degradation products.

Taken together, our findings for the first time demonstrate that C-terminal domains of Cry toxins form bona fide amyloid fibrils in vitro. Importantly, these assemblies are not biologically inert, as evidenced by their reproducible effects on cellular MTT assay readouts, indicating interaction with living cells; however the precise nature of their interaction with cells remains to be determined. Overall, this work expands current understanding of Cry toxin structural plasticity and highlights the potential functional relevance of Cry toxin fragments naturally generated by proteolysis in the insect midgut.

## 4. Materials and Methods

### 4.1. Bioinformatic Analysis of Cry Sequences

For global characterization of Cry toxins, we retrieved sequences from the BPPRC (Bacterial Pesticidal Protein Resource Center) resource [36] and the GenBank non-redundant database [37] (accessed on 15 January 2025). Cry toxins and their domain mappings were retrieved with CryProcessor v1.0 [59]. Potentially amyloidogenic regions were predicted using Waltz [38,39] and SARP [40] utilities in the sensitive mode. The identified regions were merged and classified according to the domain composition determined by CryProcessor. The distribution of sites was visualized with ggplot2 v4.0.1 [60]. Three-dimensional models were generated using ColabFold v1.5.5 [61] specifying five recycles. We then obtained the visual representation of the colored models with PyMOL v2.6 [62].

### 4.2. Bacterial Strains, DNA Isolation, and Plasmid Construction

The *B. thuringiensis* var. *darmstadiensis* strain 109/25 [63] was used as the source of genomic DNA for PCR amplification of the sequence encoding Cry1Ea11C, while the *B. thuringiensis* var. *thuringiensis* strain 800/15 [64] genomic DNA was used for the Cry1Ab12C-encoding sequence amplification. Both strains were obtained from the Russian Collection of Agricultural Microorganisms (RCAM) at the All-Russia Research Institute for Agricultural Microbiology (St. Petersburg, Russian Federation). Bacteria were cultured on solid Spizizen medium [65] (20 g/L tryptone, 5 g/L glucose, 2 g/L (NH_4_)_2_SO_4_, 6 g/L KH_2_PO_4_, 0.2 g/L MgSO_4_*7H_2_O, 18.3 g/L K_2_HPO_4_*3H_2_O, 1 g/L Na_3_C_6_H_5_O_7_*2H_2_O) at 37 °C for 12 h. Genomic DNA was isolated using a modified chloroform-phenol extraction protocol described previously [66].

The construction of the pET20b-Cry1Ea11C plasmid for overproduction of the 6His-tagged Cry1Ea11C was performed as follows. The DNA fragment encoding 6His-N-terminally tagged Cry1Ea11C was amplified by two-step PCR using primers containing restriction sites and a sequence encoding the 6His tag (two forward primers, Cry1Ea11CF1 3′-CACCATCACCACCACCTAGCAGATGCAACATTTG-5′ and Cry1Ea11CF2 3′- TATTCATATGATGCATCACCATCACCACCAC-5′, and the same reverse primer for both steps, Cry1Ea11CR 3′- CGGACTCGAGTTATTCCTCCATAAGAAGTAATTC-5′). Amplification was performed using Tersus polymerase (Evrogen, Moscow, Russia) according to the manufacturer’s instructions. Products were verified by electrophoresis on a 1% agarose gel in 1X TAE buffer at 80 V (Bio-Rad, Hercules, CA, USA). The vector pET20b-Sup35NM [67] was used as a backbone for cloning. Vector and inserts were digested with NdeI and XhoI restriction enzymes (Thermo Fisher Scientific, Waltham, MA, USA) and subsequently ligated using T4 DNA ligase (Thermo Fisher Scientific, USA) following the manufacturer’s protocol. The ligation mixture was transformed according to [68] into chemically competent *E. coli* DH5α [69] cells.

To construct the pLATE31-Cry1Ab12C plasmid for the 6His-tagged Cry1Ab12C overproduction, one-step PCR with the Cry1Ab12CF (3′-AGAAGGAGATATAACTATGGAAGTAACCTTTGAGGCAG-5′) and Cry1Ab12CR (3′- GTGGTGGTGATGGTGATGGCCTTATTCCTCCATAAGAAGTAATTC-5′) primers was performed, and PCR-amplified fragment encoding Cry1Ab12C was inserted into the pLATE31 vector through the blunt-end ligation (aLICator Kit, Thermo Fisher Scientific, USA).

Transformed cells were selected on LB medium [70] supplemented with 100 µg/mL ampicillin, and incubated at 37 °C for 12 h. Positive clones were verified by colony PCR and Sanger sequencing. The sequencing of the constructed pET20b-Cry1Ea11C and pLATE31-Cry1Ab12C plasmids was performed on an automated CEQ 8000 sequencer (Beckman Coulter, Brea, CA, USA) using the facilities of the resource center “Genomic Technologies, Proteomics and Cell Biology” of ARRIAM.

### 4.3. Protein Production and Purification

Recombinant protein expression was performed in *E. coli* BL21 (DE3) (New England Biolabs, Ipswich, MA, USA). Cells transformed either with pET20b-Cry1Ea11C or with pLATE31-Cry1Ab12C were grown in 2xTY medium (16 g/L tryptone, 10 g/L yeast extract, 5 g/L NaCl) at 28 °C with shaking (190 rpm) supplemented with 100 µg/mL ampicillin to mid-log phase and induced with 0.1 mM IPTG for 4 h. His-tagged proteins were purified using a Ni-NTA (nitrilotriacetic acid) agarose column (Invitrogen, Carlsbad, CA, USA) according to the protocol described by [71], excluding the Q-Sepharose purification step. Proteins were subsequently concentrated via ethanol precipitation. The protein concentration was measured using a Qubit 3.0 fluorometer (Invitrogen, USA) and the Qubit Protein Assay Kit (Invitrogen, USA), following the manufacturer’s instructions. Purity and integrity were assessed by SDS-PAGE following the Laemmli method [72]. The Cry1Ea11C and Cry1Ab12C constructs contained N-terminal and C-terminal 6His-tags, respectively. While tag orientation may influence aggregation behavior, both constructs were analyzed under identical conditions to enable comparative assessment.

### 4.4. Fibrillogenesis

Purified proteins were dialyzed against a modified Tris–NaCl–EDTA (mTNE) buffer as described previously [68]. Sodium azide was then added to a final concentration of 0.001% to prevent microbial contamination. Protein samples were incubated in the mTNE buffer under continuous agitation at room temperature for one month to induce fibrillogenesis.

### 4.5. Congo Red Staining and Polarized Light Microscopy

Amyloidogenic aggregates were analyzed using Congo Red staining. Aliquots (2.5 µL) of samples were applied to glass slides and air-dried. Congo Red solution (10 mg/mL) (Sigma, USA) was applied, followed by washing with 96% ethanol. Samples were visualized using a Zeiss Axio Imager A2 microscope (Carl Zeiss, Oberkochen, Germany) fitted with a Plan-Neofluar 40× objective and cross-polarizers. Images were captured under both transmitted and polarized light conditions.

### 4.6. Thioflavin T and Amytracker Fluorescence Analysis

For Thioflavin T staining, protein samples were mixed with ThT (Thermo Scientific Chemicals, USA) solution and applied to glass slides. Amytracker™ 520 (Ebba Biotech AB, Stockholm, Sweden) staining was performed according to the manufacturer’s instructions.

Fluorescence was visualized using a Zeiss Axio Imager A2 microscope (Carl Zeiss, Germany) fitted with a Plan-Neofluar 40× objective and illuminated by a Colibri 7 (LED) fluorescence light source (Carl Zeiss, Germany). Fluorescence imaging was conducted under identical conditions to allow comparison between samples.

### 4.7. Transmission Electron Microscopy (TEM)

Protein samples (5 µL) were applied to Formvar/Carbon copper grids, 200 Mesh (Electron Microscopy Sciences, Morgantown, PA, USA) as described previously [52]. Grids were washed with deionized water and stained with 1% uranyl acetate. Images were acquired using a Libra 120 transmission electron microscope (Carl Zeiss, Germany). To determine fibril width, we analyzed a total of 80 individual fibrils from 12 independent TEM images acquired across 3 biological replicates. The measurements were performed using ImageJ/Fiji software (version 1.52i).

### 4.8. X-Ray Diffraction Analysis

Fibrillar samples were mounted between wax-coated glass capillaries (approximately 1 mm in diameter) positioned 1.5 mm apart. X-ray diffraction patterns were collected using a Rigaku XtaLAB Synergy-S single-crystal X-ray diffractometer (Rigaku Corporation, Tokyo, Japan) equipped with a Hypix-6000C detector using the CrysAlisPro software, version 1.171.42.89a (Rigaku, Tokyo, Japan).

### 4.9. SDS and Protease Resistance Assays

Aggregated protein samples were analyzed by SDS-PAGE, following the Laemmli method [72], under both non-boiled and boiled (7 min) conditions. For protease digestion, samples were treated with Trypsin (Sigma-Aldrich, Saint-Louis, MO, USA) or Proteinase K (Evrogen, Moscow, Russia) at 1:60 and 1:30 to the total protein mass ratio, respectively, followed by incubation at 37 C for 15 min. Reactions were stopped by addition of SDS sample buffer, followed by electrophoretic analysis. The analysis was conducted using the TGX Stain-Free FastCast acrylamide gel kit (Bio-Rad), with the PageRuler Plus Prestained Ladder (Thermo Scientific) as the molecular weight marker. Protein electrophoresis was carried out for 45 min at 180 V. Detection of the results was performed using the ChemiDoc XRS+ System (Bio-Rad). Coomassie Brilliant Blue R-250 (Bio-Rad) staining was performed according to the manufacturer’s instructions.

### 4.10. MTT Assay

The cytotoxic effects of protein samples on Sf21 cells (IPLB-Sf21-AE) (Cat. No. B82101) from *Spodoptera frugiperda* (Fall Armyworm) were evaluated as follows: the cells were cultured in 96-well plates with 100 μL of Serum-free ESF921 medium (Expression Systems #96-001-01, Davis, CA, USA) supplemented with 100 U/mL penicillin, 100 μg/mL streptomycin, 50 μg/mL gentamicin, and 10% sample buffer at a seeding density of 5 × 10^5^ cells/mL. Control wells received buffer without protein. The fibrillar and non-fibrillar samples were tested at final concentrations of 0.025, 0.05, and 0.075 mg/mL. After incubation at 28 °C for 24 h, cell viability was assessed via MTT assay [45]. Briefly, 10 μL of MTT solution (5 mg/mL in PBS) was added to each well and incubated at 28 °C for 4 h. Following this, 100 μL of SDS-HCl solution (10% SDS, 0.01 N HCl) was added, and the plates were incubated for an additional 16 h. Absorbance was recorded at 570 nm with background subtraction at 620 nm.

All experiments were conducted in quadruplicate. Statistical analysis was performed using one-way analysis of variance (ANOVA) followed by the emmeans post hoc test (emmeans R package, https://github.com/rvlenth/emmeans; accessed on 10 August 2023). Differences were considered statistically significant at *p* < 0.05.

### 4.11. Trypan Blue Dye Exclusion Method

SF21 cells were seeded in 96-well plates at a density of 45,000 cells per well (90 µL of 500,000 cells/mL suspension) and incubated with 10 µL of each protein sample (1 mg/mL stock, final concentration 50 µg/mL) or with buffer alone (control) for 24 h at 28 °C. Following incubation, cell viability was determined by the Trypan Blue dye exclusion method. Briefly, 10 µL of cell suspension was mixed with 10 µL of 0.4% Trypan Blue Solution (Gibco™, Thermo Fisher Scientific). A total of 10 µL of the mixture was loaded into a cell counting chamber (Minimed, Pune, India). Viable (unstained) and non-viable (blue-stained) cells were enumerated in 27 small squares per replicate. All experiments were conducted in quadruplicate.

## Figures and Tables

**Figure 1:**
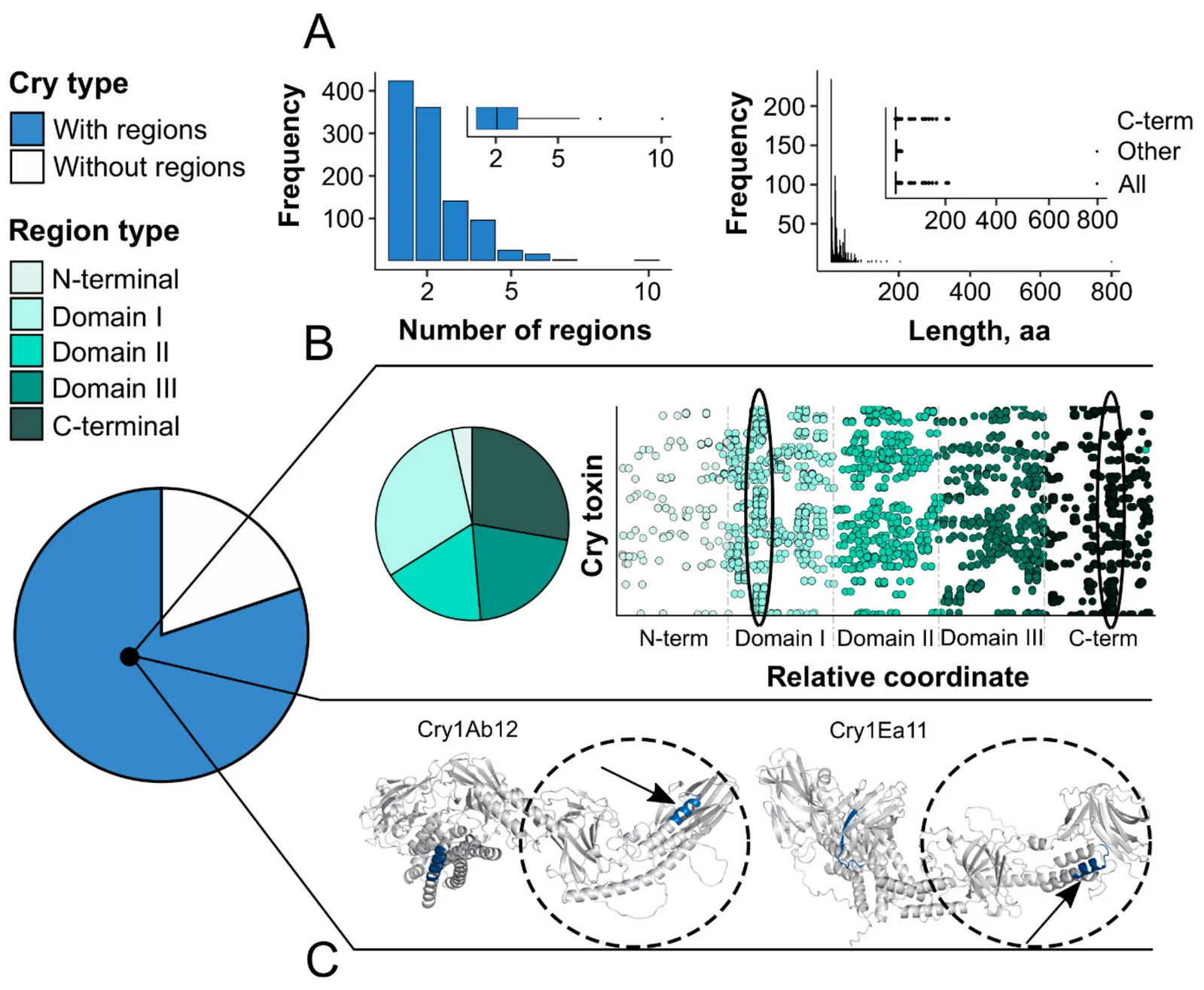
Predicted amyloidogenic landscape of Cry toxins. (**A**) The common properties of computationally predicted amyloidogenic regions. Displayed plots are the number of such sites and the total length of all putative amyloidogenic zones. (**B**) Structure-wise classification of the revealed regions. The relative coordinates of start and end positions of the results, coupled with the total distribution, are given. The black dotted ellipses point to the small zones with conserved position among Cry sequences. Colores correspond to the domain to which the amyloidogenic signal was attributed. (**C**) Reconstructed three-dimensional structures of the studied proteins Cry1Ab12 and Cry1Ea11. Colorized parts designate potential amyloidogenic sites. The black circles illustrate C-terminal domains, while black arrows point to the amyloidogenic regions within these domains.

**Figure 2:**
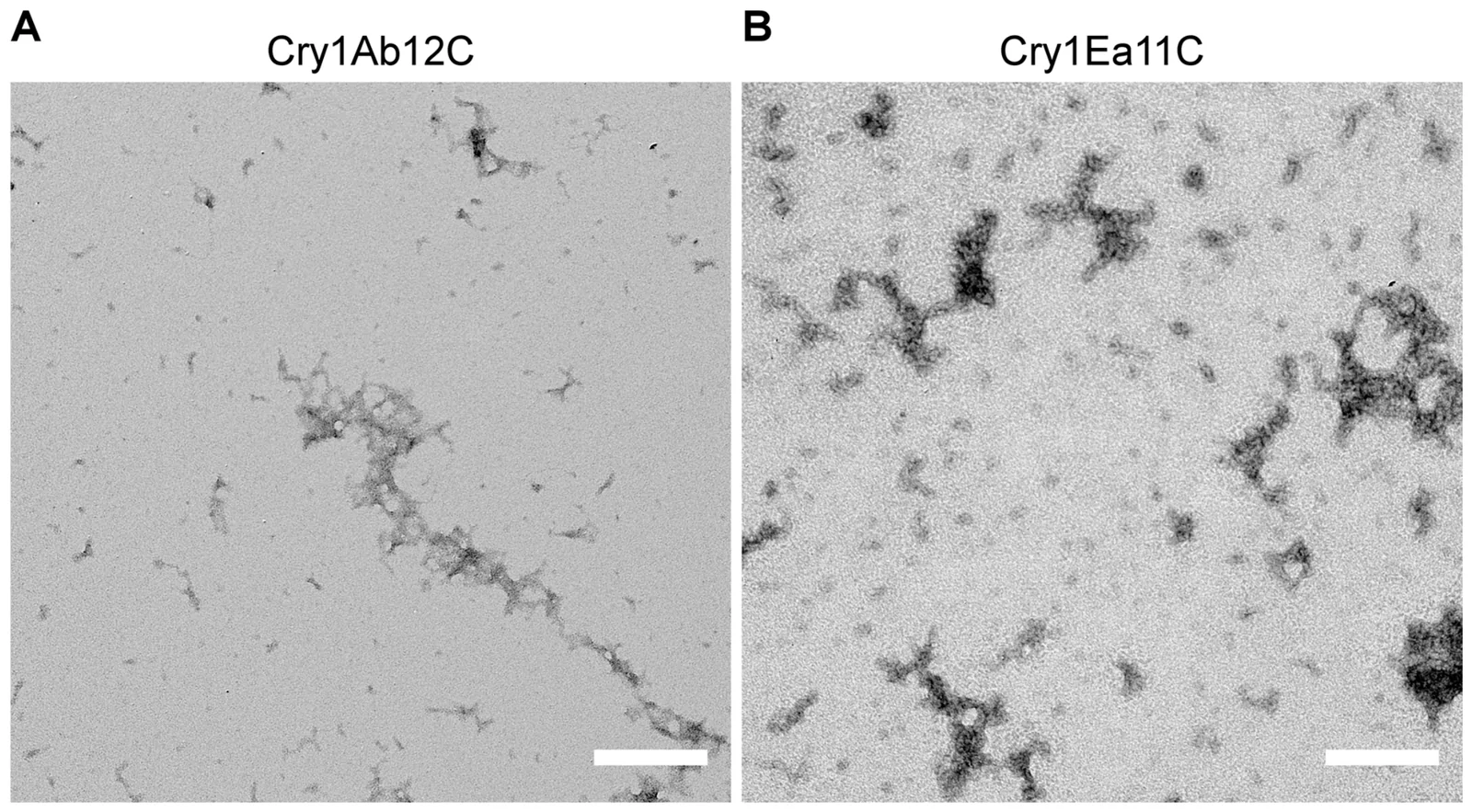
Visualization of fibrillar aggregates formed from the C-terminal domains of Cry toxins using transmission electron microscopy (TEM). TEM images of the aggregates formed from Cry1Ab12C (**A**) and Cry1Ea11C (**B**). Scale bars: 200 nm (applies to all panels).

**Figure 3:**
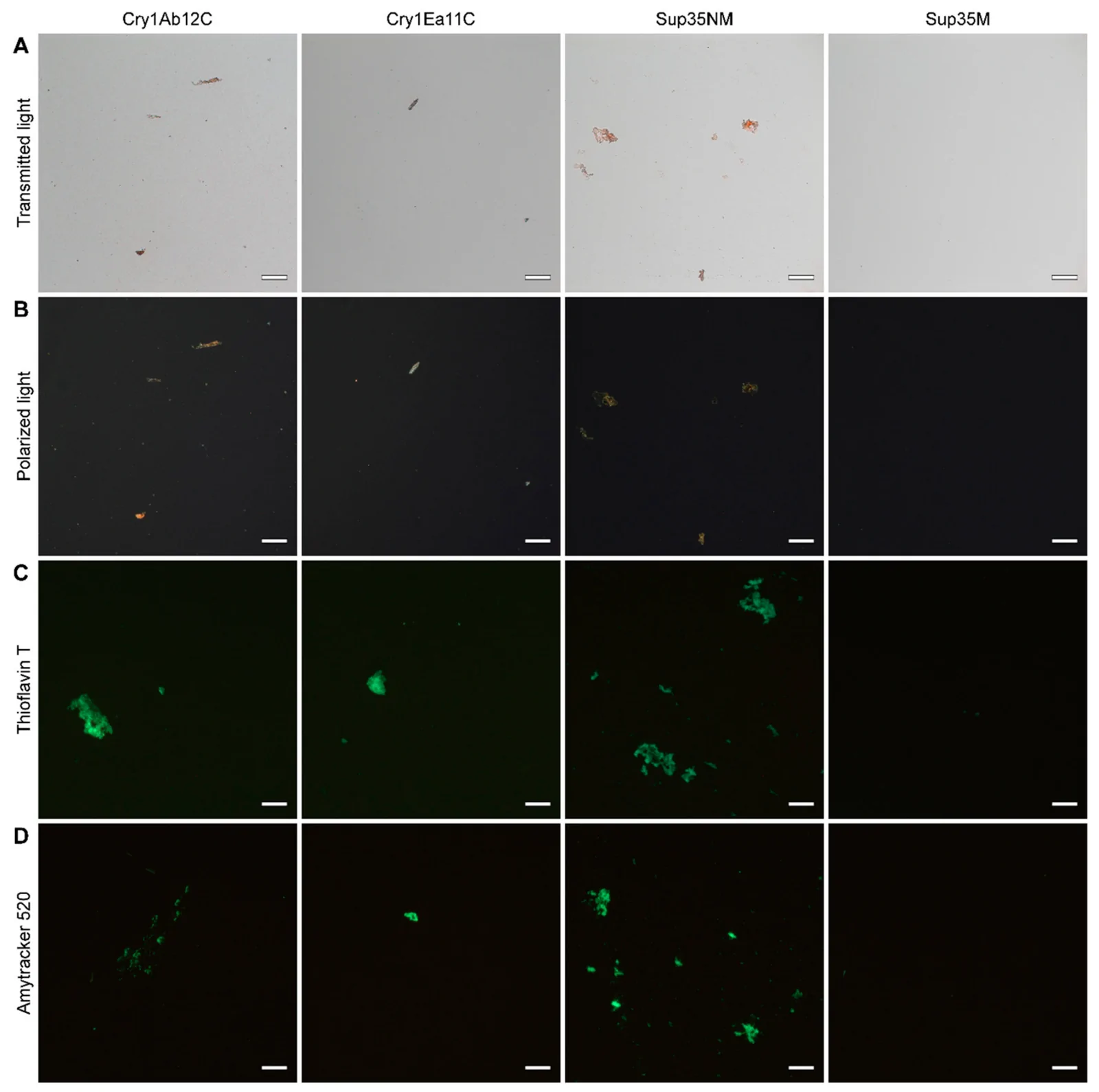
Interaction of aggregates formed from the C-terminal domains of Cry toxins with CR, ThT and Amytracker dyes. Polarized light microscopy of the aggregates stained with CR under transmitted light (**A**) and polarized light (**B**). (**C**) Fluorescent microscopy of the aggregates stained with ThT. (**D**) Fluorescent microscopy of the aggregates stained with Amytracker. The Sup35NM (amyloid-positive control) and Sup35M (soluble, amyloid-negative control) proteins were used as indicated. Scale bar: 50 μm (applies to all panels).

**Figure 4:**
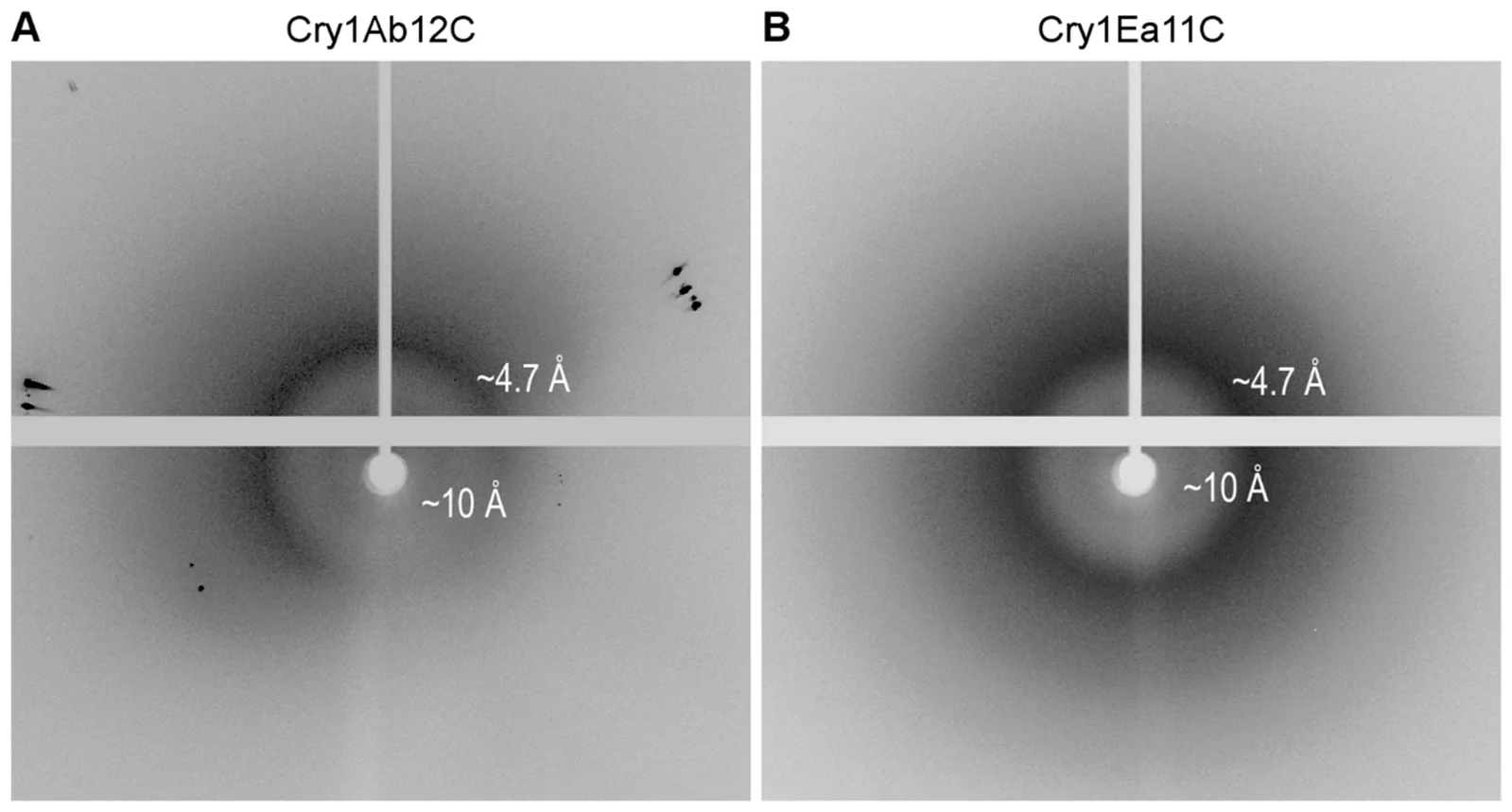
Analysis of the structure of in vitro-obtained aggregates by X-ray diffraction. X-ray diffraction patterns of lyophilized Cry1Ab12C (**A**) and Cry1Ea11C (**B**). Reflections are labeled in angstroms (Å).

**Figure 5:**
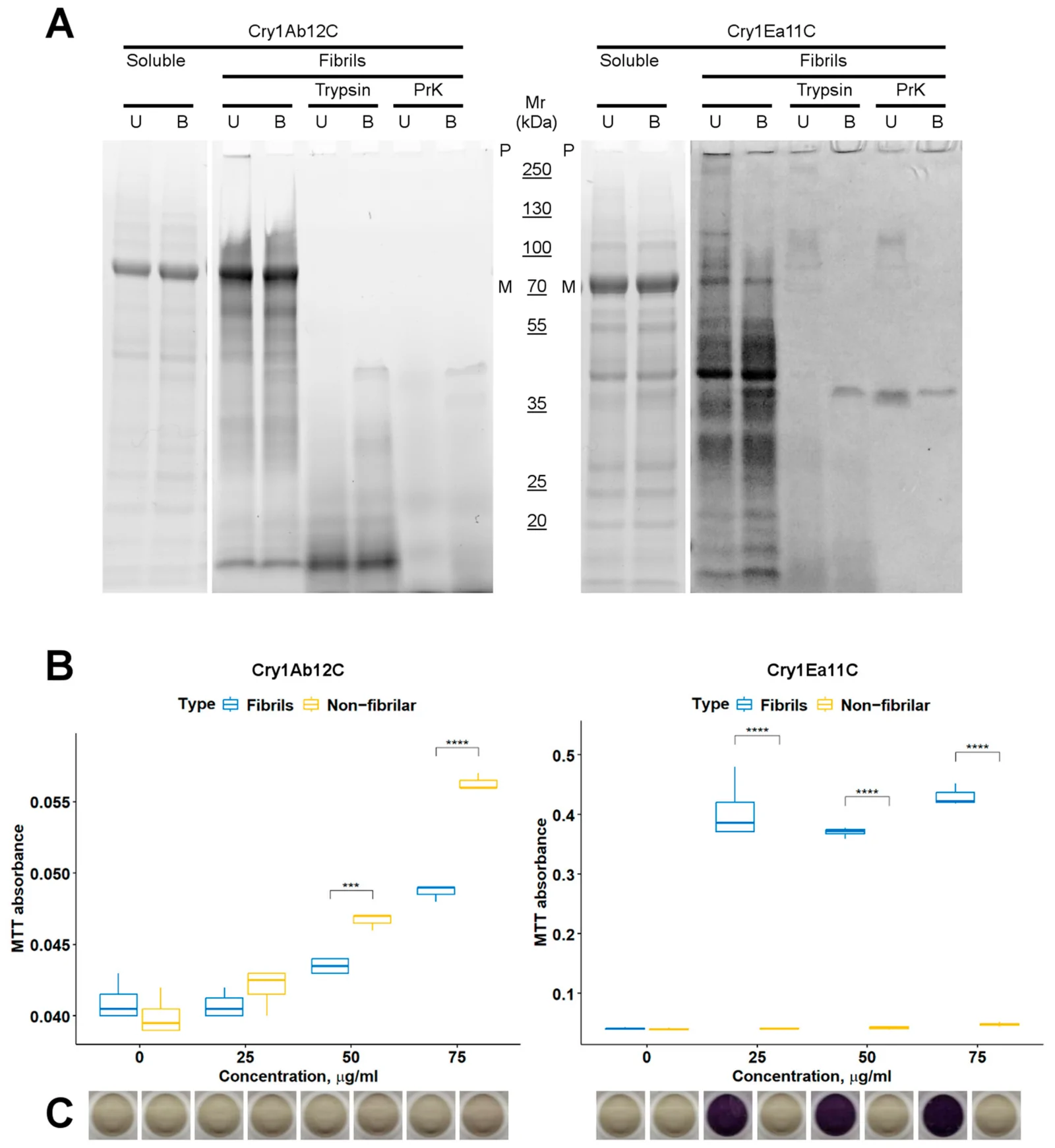
Biochemical properties of Cry toxin C-terminal domain aggregates. (**A**) Resistance of aggregates of the Cry C-terminal domains to treatment with SDS and proteases. Aggregates were treated with 2% SDS at room temperature (U, unboiled) or after boiling (B, boiled), or with trypsin or proteinase K (PrK). P: Polymers. M: Monomers. Molecular weights (kDa) are shown. (**B**) Quantification of MTT reduction in Sf21 cells exposed to different concentrations of Cry1Ab12C (left panel) and Cry1Ea11C (right panel) proteins. Color denotes protein form. Data are presented as box plots, and whiskers indicate the data range. *** *p* ≤ 0.001, **** *p* ≤ 0.0001. (**C**) Representative images of wells from a 96-well MTT assay plate. Each condition was analyzed in quadruplicate.

## Data Availability

The original contributions presented in this study are included in the article. Further inquiries can be directed to the corresponding author.
